# Molecular Interactions in the Development of Brain Metastases

**DOI:** 10.3390/ijms140817157

**Published:** 2013-08-20

**Authors:** Nina Martinez, Adrienne Boire, Lisa M. DeAngelis

**Affiliations:** Department of Neurology, Memorial Sloan-Kettering Cancer Center, 1275 York Avenue, New York, NY 10065, USA; E-Mails: nlmartinez@gmail.com (N.M.); boirea@mskcc.org (A.B.)

**Keywords:** CNS metastasis, metastatic cascade, vascular co-option, dormancy

## Abstract

Brain metastases are a much-feared complication of cancer. The development of brain metastases requires a malignant cell to acquire characteristics that facilitate dissemination away from the primary site, entrance into the nervous system, and establishment in the brain. This review summarizes recent work focused on the molecular derangements leading to brain metastases and outlines areas in need of greater understanding.

## 1. Introduction

The dissemination and growth of tumor cells distant from their site of origin is a much-feared complication of cancer. Brain metastases are particularly devastating due to their neurocognitive impact and resistance to conventional therapy, and are often perceived as a marker of end-stage cancer and imminent death. Over 150,000 patients are diagnosed with brain metastases each year in the United States, which is an increase compared to a previously reported incidence of 17,380 three decades ago [1,2]. This may be due to various reasons, including improvements in diagnostic techniques such as imaging. The most common solid tumors which spread to the brain primarily include those of lung, breast, and melanoma origin, though a recent increase in the incidence of brain metastases from other cancer types such as renal, prostate, and colorectal has been observed [3–5]. The median survival of untreated patients is only 1–2 months, which is only marginally extended to 6 months after surgery and radiation [6–10]. A small subset of patents may benefit from chemotherapy, though overall response remains poor [11–15]. Understanding the metastatic process is a key step to the development of new therapeutics and improvement of patient outcomes.

More than 100 years ago, Paget’s “seed and soil” hypothesis—which proposes that the basis of metastasis involves a favorable interaction between a cancer cell (the “seed”) and an organ microenvironment (the “soil”)—established the fundamental relationship between the tumor cell and a secondary organ to support the establishment and growth of a metastasis. The process resulting in the distant colonization of tumor cells into a secondary organ is a consequence of multiple interrelated events that is classically referred to as the “metastatic cascade.” Traits that increase a tumor cell’s potential to access and establish itself in the brain and other distant organs can be acquired during this process. Earlier models depict this process as a unidirectional sequence of events—oncogenic transformation, local invasion, intravasation, survival in the circulation, extravasation, and colonization. Though the order in which the steps occur may vary among tumor types, each step is pertinent to determining a tumor cell’s fate and can be interrupted at any time by homeostatic mechanisms [16–18].

Before reaching the central nervous system (CNS), cells of the primary tumor first acquire mutations that lead to genetic instability, sustained proliferative signaling, evasion of growth suppressors, increased invasive potential, replicative immortality, resistance to hypoxia and cell death, and some develop stem-like capabilities. Recent work suggests tumor cells that have already spread to secondary sites can reinfiltrate and colonize the primary tumor, interact with the microenvironment, acquire new genetic signatures that breed more aggressive phenotypes, and proceed to seed and colonize distant organs again [18–21]. This process is known as “self-seeding” and has been demonstrated in animal models to occur with pulmonary metastases but has not yet been shown to occur with brain metastases. However, this process results in cells with enhanced propensity to grow and spread distantly and this may well affect the brain. Early research efforts focused on the properties of the cancer cell, but recent work suggests that the microenvironment is critically important in the metastatic process [22,23]. In this review, we discuss the known molecular interactions leading to brain metastasis and describe areas in need of further investigation.

## 2. Development of Brain Metastases

### 2.1. The BBB: Normal Structure and Function

The blood brain barrier (BBB) can be defined anatomically by the neurovascular unit, which consists of endothelial cells, pericytes, and astrocytes. These cells function as a highly regulated unit to control blood flow in response to neural demand at the micro-anatomic level. Since the brain does not contain lymphatics, circulating tumor cells reach the brain parenchyma only via a hematogenous route. Unlike capillary structures in the majority of the body, the cerebral vasculature is unique in that continuous tight junctions and low pinocytic activity at the capillary level restrict the entrance of most macromolecules from the blood into the CNS.

The tight junctions between capillary endothelial cells are composed of intricate links between various transmembrane and cytoplasmic proteins; three integral proteins—claudin, occludin, and junctional adhesion molecule—have been identified and together they form a seal, which permits free transport of only small solutes [24]. Pericytes found upon the basal surface of the capillary endothelial cell have been demonstrated *in vivo* to regulate the water permeability across the BBB and are implicated in mediation of astrocyte end-feet attachment to the abluminal vessel surface [25]. Astrocytes are important in that they may regulate expression of proteins such as P-glycoprotein (P-gp), aquaporin-4 (AQP-4), and glucose-transporter 1 (GLUT-1) which are involved in the transport of various substances across the BBB. Furthermore, astrocytes have the capacity to secrete a range of chemical factors in response to stress such as stroke, trauma, or inflammation, and may play a critical role in the invasion of tumor cells into the brain [26–28].

### 2.2. Invasion and Manipulation of the BBB

There is evidence that invading metastatic cancer cells interact with all of these cell types—endothelium, pericytes, and astrocytes—to breach the BBB and gain access to the brain parenchyma [29]. How some circulating tumor cells are able to invade the BBB is not fully understood, but several key interactions have been identified through the use of multiphoton laser scanning microscopy [30]. Single cancer cells or heterotypic clusters of viable tumor mixed with dead cells or blood components arrest at vascular branch points, where blood flow is reduced. Following adhesion, the cancer cell may begin to interact with the cerebral vasculature. Some of the key mediators involved in this process have been identified and are summarized in Table 1.

In the breast cell line MDA-MB-435, cells adapt to the narrow cerebral capillaries by reshaping themselves into an elongated form until just before or during extravasation. This process involved expansion of the surrounding vessel wall by amassing and extending tumor cytoplasmic projections to penetrate the blood vessel wall. Although disruption of the vessel wall occurred, no significant destruction of the vascular endothelium was identified [31]. This mechanism is known as vascular co-option, and *in vitro* experiments on human breast carcinoma cell line MDA-MB-231 have identified tumor cell β1 integrins as essential to the adhesion of tumor cells to the vascular basement membrane [32]. Cell-to-cell interactions enhanced by catalysis of cell-surface proteins by sialyltransferases such as ST6GALNAC5 (observed *in vivo* with brain-tropic breast cancer cell line MDA231) may also facilitate tumor-endothelium adhesion [30,34].

The secretion of proteolytic enzymes (e.g., matrix metallopeptidase 9 and heparanase), cytokines (e.g., transforming growth factor β2), and chemokines (e.g., stromal cell-derived factor 1α) have been associated with the extravasation process in other cancer cell lines, and implies variation in the manner in which tumor cells exit capillary channels [36]. In brain-metastatic human melanoma cell lines, neurotrophins enhance production of heparanase, an enzyme which degrades the extracellular matrix and basement membrane that characterize the BBB [37].

Once tumor cells enter the brain parenchyma, a number of factors are released by the tumor cells and underlying brain. In response to tumor-associated migration inhibitory factor (MIF), interleukin-8 (IL-8), and plasminogen activator inhibitor-1 (PAI-1), astrocytes release various inflammatory cytokines including IL-1β, TNF-α, and IL-6, all of which have been shown to promote tumor cell proliferation *in vitro* [38–42].

### 2.3. Chemoprotection by the Brain Microenvironment

Some patients develop new brain metastases while their systemic disease is responding to chemotherapy, which implies that the CNS may serve as a sanctuary site for some tumor cells. This is exemplified in patients who receive trastuzumab for HER-2 positive breast cancer and those receiving gefitinib for EGFR-mutant lung adenocarcinoma [43,44]. One explanation is that the selective pressures in the CNS vary from those in other organs to permit the survival of cells that have acquired favorable mutations. Another hypothesis is that the targeting drug penetrates poorly into the CNS [45]. These mechanisms are not mutually exclusive but inadequate drug penetration is at least a contributing factor because once the serum level of chemotherapy is sufficiently high to achieve therapeutic levels in the CNS, then brain metastases may respond [46,47].

The penetration of large molecule therapeutics through the BBB is one obstacle to the effective treatment of CNS tumors with systemic agents, though the process may be more active than previously thought. A recent *in vitro* study suggests that chemoprotection against paclitaxel is an active process exploiting the actions of activated astrocytes. In response to direct contact with human breast or lung tumor cells, murine astrocytes induce upregulation of survival genes in the cancer cell such as GSTA5, BCL2L1, and TWIST1 [47,48]. These genes are associated with drug resistance, evasion of apoptosis, and survival, and upregulation was shown to be a downstream effect of AKT and mitogen-activated protein kinase (MAPK) activation. These animal models afford the opportunity to clarify this pathway further.

A potential method of circumventing the physical barrier of the BBB is to combine a cytotoxic agent with an efflux pump inhibitor. In one experiment, paclitaxel-sensitive human melanoma cells (K1735) were implanted subcutaneously and intracerebrally into immunodeficient mice. While the subcutaneous tumors regressed after administration of paclitaxel, the intracerebral tumors did not. This resistance was overcome by combining paclitaxel with HM30181A, a P-gp inhibitor, thus facilitating drug entry into the brain [49]. This finding provides critical information about the role of efflux pumps as potential therapeutic targets and demonstrates the potential clinical importance of developing more effective efflux pump inhibitors.

### 2.4. Dormancy

The long latency between the successful treatment of systemic disease and the development of brain macrometastases suggests that tumor cells might exist within the CNS inactive for some time. This phenomenon has been demonstrated in experimental animals by injecting tagged 231-BR human breast cancer cells expressing green fluorescent protein with micron-sized iron oxide particles, making the cells detectable on MRI. The animals were then monitored with serial brain MRIs for evidence of CNS dissemination. Cells that actively divided lost ferric signal but retained fluorescence; 94% of the original cells could not be detected on MRI, 1.6% developed into macrometastases, and 4.5% retained ferric signal but did not proliferate [50]. The static nature of this third group suggests a state of dormancy, which may be a survival strategy for cells that are unable to proliferate yet manage to avoid apoptosis [51–56].

The mechanisms that drive tumor cell dormancy are still unknown, but current research may shed more light on this and provide a pathway for prevention or treatment of brain metastases [57]. Three underlying hypotheses, which are not mutually exclusive, have been proposed [58]. The first proposes that once a disseminated tumor cell reaches a distant organ, a host response is triggered. The stress signals released by the host organ activate the p38 pathway, which inhibits cell proliferation but does not induce death [59,60]. The second is based upon the concept of self-seeding, in which a disseminated tumor cell returns to the primary organ, interacts with the microenvironment, and acquires new gene signatures that induce quiescence in response to host signaling [57,61]. The third proposes that tumor cells are able to invade into and arrest within a target organ, but require further epigenetic or genetic changes that must be acquired within the secondary site in order to proliferate [62].

Genetic signatures responsible for inducing quiescence are referred to as metastasis suppressor genes (MSG). The earliest MSG was identified in 1988 through differential gene expression of cell lines derived from K-1375 melanoma [63,64]. The expression of cDNA non-metastatic 23 (nm23) was downregulated in highly metastatic cell lines as compared to two related, less metastatic lineages. Since then, a multitude of MSGs have been identified including KAI1, KISS1, and MKK4/7. By acting on signal transduction pathways such as the MAPK/extracellular signal regulated kinase (ERK), stress-activated protein kinase/c-Jun *N*-terminal kinase (SAPK/JNK) or p38, these genes can induce growth arrest at metastatic sites without initiating apoptosis. These genes could be critical to maintaining dormancy for prolonged periods of time, even if tumor cells have gained access and survive within a secondary organ. Further understanding of the early changes that occur in the primary tumor and events in the target organ that trigger the expression of MSGs may provide new therapeutic targets to suppress brain metastasis growth.

## 3. Genetic Alterations Associated with Increased Brain Metastatic Potential

Enhanced potential for the development of CNS metastases may be identified in the primary and define future therapeutic targets. For example, overexpression of human epidermal growth factor receptor 2 (HER2/neu) is predictive of a 3-fold increase in metastases to the lungs, liver, and brain compared to HER2/neu negative breast carcinomas [65–69]. This discovery has prompted clinical trials for this subset of patients [70–72]. In lung adenocarcinoma, genetic alterations in HOXB9 and LEF1 lead to hyperactivity of the WNT/TCF pathway, which has been implicated in the growth of cancer stem cells and enhanced competence to metastasize to the bone and brain [69,73]. How overexpression of *HOXB9* and *LEF1* alters the interactions among tumor cell, pressures within the circulation, and brain endothelium is currently unknown.

Other recently described gene expression signatures may predict outcome such as the risk of organ-specific dissemination [32,74]. For example, comparative genome-wide expression analysis on a breast cancer cell line with preference for migration to the brain identified three genes that mediate tumor cell passage through the blood brain barrier (BBB): COX2, HBEGF, and ST6GALNAC5. Activation of COX2 and HBEGF were also associated with pulmonary metastases which is interesting as more than 70% of patients with brain metastases from non-pulmonary primaries have co-existent lung metastases. As discussed previously, ST6GALNAC5 was observed only in the cell line associated with brain-tropism and appears to be integral to cell extravasation out of the vasculature and into the brain tissue [28]. These findings are only the earliest indicators of the underlying biology that defines brain tropism for some metastatic clones. Furthermore, the self-seeding phenomenon indicates that this is an on-going, active process and not a static phenomenon. This likely plays a role not only in the initial development of brain metastases but also for their recurrence, both at an original site and elsewhere in the brain, after therapy. The hope is that delineation of these mechanisms will provide novel therapeutic targets for both the prevention and treatment of brain metastases.

## 4. Conclusions

The pathophysiology of brain metastasis is complex and dependent upon both oncogenic processes and host organ responses. Multiple mechanisms determine the ultimate development of a brain metastasis including but not limited to brain-trophic tumor cell phenotypes, tumor cell survival in the vasculature and extravasation of those cells from the bloodstream and into a host organ, and the structure and function of the BBB. Many unanswered questions remain, including the molecular basis of brain-tropism and the signals that govern dormancy of tumor cells and their subsequent proliferation after a period of quiescence. Future work will hopefully identify therapeutic targets that will prevent brain metastases from taking hold or treat established CNS metastases.

## Figures and Tables

**Table 1:** Known mediators involved in tumor cell-blood brain barrier (BBB) interaction.

Mediator	Action	Primary tumor	Reference
Stromal cell-derived factor 1α	Adhesion; tumor cell migration	MDA-MB231, DU4475 (breast)	Lee, 2004 [30]
β1 integrins	Tumor cell adhesion to endothelial cell basement membrane	MDA-MB231 (breast)	Carbonell, 2009 [31]
ST6GALNAC5	Tumor cell adhesion to endothelial cell basement membrane	MDA-MB231 (breast)	Bos, 2009 [32]
Heparanase	Proteolysis	70W (melanoma)	Marchetti, 2000 [26]
Matrix metalloproteinases	Invasion; mechanism unknown	ENU1564 (breast)	Mendes, 2005 [33]
TGF-β2	Growth factor	K-1735 (melanoma)	Zhang, 2009 [34]
IL-6, IGF-1	Growth factor	MDA-MB435 (breast)	Sierra, 1997 [35]

## References

[1] Walker A.E., Robins M., Weinfeld F.D. (1985). Epidemiology of brain tumors: The national survey of intracranial neoplasms. Neurology.

[2] Ewend M.G., Morris D.E., Carey L.A., Ladha A.M., Brem S. (2008). Guidelines for the initial management of metastatic brain tumors: Role of surgery, radiosurgery, and radiation therapy. J. Natl. Compr. CancER Netw.

[3] Barnholtz-Sloan J.S., Sloan A.E., Davis F.G., Vigneau F.D., Lai P., Sawaya R.E. (2004). Incidence proportions of brain metastases in patients diagnosed (1973 to 2001) in the metropolitan detroit cancer surveillance system. J. Clin. Oncol.

[4] Fox B.D., Cheung V.J., Patel A.J., Suki D., Rao G. (2011). Epidemiology of metastatic brain tumors. Neurosurg. Clin. N. Am.

[5] Davis F.G., Dolecek T.A., McCarthy B.J., Villano J.L. (2012). Toward determining the lifetime occurrence of metastatic brain tumors estimated from 2007 united states cancer incidence data. Neuro Oncol.

[6] Patchell R.A., Tibbs P.A., Walsh J.W., Dempsey R.J., Maruyama Y., Kryscio R.J., Markesbery W.R., Macdonald J.S., Young B. (1990). A randomized trial of surgery in the treatment of single metastases to the brain. N. Engl. J. Med.

[7] Patchell R.A. (2003). The management of brain metastases. Cancer Treatment Rev.

[8] Andrews D.W., Scott C.B., Sperduto P.W., Flanders A.E., Gaspar L.E., Schell M.C., Werner-Wasik M., Demas W., Ryu J., Bahary J.P. (2004). Whole brain radiation therapy with or without stereotactic radiosurgery boost for patients with one to three brain metastases: Phase iii results of the rtog 9508 randomised trial. Lancet.

[9] Aoyama H., Shirato H., Tago M., Nakagawa K., Toyoda T., Hatano K., Kenjyo M., Oya N., Hirota S., Shioura H. (2006). Stereotactic radiosurgery plus whole-brain radiation therapy *vs*. stereotactic radiosurgery alone for treatment of brain metastases: A randomized controlled trial. JAMA.

[10] Eichler A.F., Loeffler J.S. (2007). Multidisciplinary management of brain metastases. Oncologist.

[11] Ceresoli G.L., Cappuzzo F., Gregorc V., Bartolini S., Crino L., Villa E. (2004). Gefitinib in patients with brain metastases from non-small-cell lung cancer: A prospective trial. Ann. Oncol.

[12] Bartolotti M., Franceschi E., Brandes A.A. (2012). Egf receptor tyrosine kinase inhibitors in the treatment of brain metastases from non-small-cell lung cancer. Expert Rev. Anticancer Ther.

[13] Rochet N.M., Dronca R.S., Kottschade L.A., Chavan R.N., Gorman B., Gilbertson J.R., Markovic S.N. (2012). Melanoma brain metastases and vemurafenib: Need for further investigation. Mayo Clin. Proc.

[14] Soffietti R., Trevisan E., Ruda R. (2012). Targeted therapy in brain metastasis. Curr. Opin. Oncol.

[15] Mehta M.P., Paleologos N.A., Mikkelsen T., Robinson P.D., Ammirati M., Andrews D.W., Asher A.L., Burri S.H., Cobbs C.S., Gaspar L.E. (2010). The role of chemotherapy in the management of newly diagnosed brain metastases: A systematic review and evidence-based clinical practice guideline. J. Neurooncol.

[16] Gupta G.P., Massague J. (2006). Cancer metastasis: Building a framework. Cell.

[17] Chambers A.F., Groom A.C., MacDonald I.C. (2002). Dissemination and growth of cancer cells in metastatic sites. Nat. Rev. Cancer.

[18] Langley R.R., Fidler I.J. (2011). The seed and soil hypothesis revisited—The role of tumor-stroma interactions in metastasis to different organs. Int. J. Cancer.

[19] Fidler I.J., Hart I.R. (1982). Biological diversity in metastatic neoplasms: Origins and implications. Science.

[20] Zhang C., Yu D. (2011). Microenvironment determinants of brain metastasis. Cell Biosci.

[21] Fidler I.J. (2011). The role of the organ microenvironment in brain metastasis. Semin. Cancer Biol.

[22] Kniesel U., Wolburg H. (2000). Tight junctions of the blood-brain barrier. Cell Mol. Neurobiol.

[23] Huber J.D., Egleton R.D., Davis T.P. (2001). Molecular physiology and pathophysiology of tight junctions in the blood-brain barrier. Trends Neurosci.

[24] Armulik A., Genove G., Mae M., Nisancioglu M.H., Wallgard E., Niaudet C., He L., Norlin J., Lindblom P., Strittmatter K. (2010). Pericytes regulate the blood-brain barrier. Nature.

[25] Abbott N.J., Ronnback L., Hansson E. (2006). Astrocyte-endothelial interactions at the blood-brain barrier. Nat. Rev. Neurosci.

[26] Marchetti D.L.J., Shen R. (2000). Astrocytes contribute to the brain-metastatic specificity of melanoma cells by producing heparanase. Cancer Res.

[27] Lorger M., Felding-Habermann B. (2010). Capturing changes in the brain microenvironment during initial steps of breast cancer brain metastasis. Am. J. Pathol.

[28] Lorger M., Lee H., Forsyth J.S., Felding-Habermann B. (2011). Comparison of *in vitro* and *in vivo* approaches to studying brain colonization by breast cancer cells. J. Neurooncol.

[29] Kienast Y., von Baumgarten L., Fuhrmann M., Klinkert W.E., Goldbrunner R., Herms J., Winkler F. (2010). Real-time imaging reveals the single steps of brain metastasis formation. Nat. Med.

[30] Lee B., Lee T., Avraham S., Avraham H. (2004). Involvement of the chemokine receptor cxcr4 and its ligand stromal cell-derived factor 1alpha in breast cancer cell migration through human brain microvascular endothelial cells. Mol. Cancer Res.

[31] Carbonell W.S., Ansorge O., Sibson N., Muschel R. (2009). The vascular basement membrane as “soil” in brain metastasis. PLoS One.

[32] Bos P.D., Zhang X.H., Nadal C., Shu W., Gomis R.R., Nguyen D.X., Minn A.J., van de Vijver M.J., Gerald W.L., Foekens J.A. (2009). Genes that mediate breast cancer metastasis to the brain. Nature.

[33] Mendes O., Kim H.T., Stoica G. (2005). Expression of mmp2, mmp9 and mmp3 in breast cancer brain metastasis in a rat model. Clin. Exp. Metast.

[34] Zhang C., Zhang F., Tsan R., Fidler I.J. (2009). Transforming growth factor-beta2 is a molecular determinant for site-specific melanoma metastasis in the brain. Cancer Res.

[35] Sierra A., Price J.E., Garcia-Ramirez M., Mendez O., Lopez L., Fabra A. (1997). Astrocyte-derived cytokines contribute to the metastatic brain specificity of breast cancer cells. Lab. Invest.

[36] Denkins Y., Reiland J., Roy M., Sinnappah-Kang N.D., Galjour J., Murry B.P., Blust J., Aucoin R., Marchetti D. (2004). Brain metastases in melanoma: Roles of neurotrophins. Neuro Oncol.

[37] Seike T., Fujita K., Yamakawa Y., Kido M.A., Takiguchi S., Teramoto N., Iguchi H., Noda M. (2011). Interaction between lung cancer cells and astrocytes via specific inflammatory cytokines in the microenvironment of brain metastasis. Clin. Exp. Metast.

[38] Bendell J.C., Domchek S.M., Burstein H.J., Harris L., Younger J., Kuter I., Bunnell C., Rue M., Gelman R., Winer E. (2003). Central nervous system metastases in women who receive trastuzumab-based therapy for metastatic breast carcinoma. Cancer.

[39] Stemmler H.J., Kahlert S., Siekiera W., Untch M., Heinrich B., Heinemann V. (2006). Characteristics of patients with brain metastases receiving trastuzumab for her2 overexpressing metastatic breast cancer. Breast.

[40] Lower E.E., Drosick D.R., Blau R., Brennan L., Danneman W., Hawley D.K. (2003). Increased rate of brain metastasis with trastuzumab therapy not associated with impaired survival. Clin. Breast Cancer.

[41] Yau T., Swanton C., Chua S., Sue A., Walsh G., Rostom A., Johnston S.R., O’Brien M.E., Smith I.E. (2006). Incidence, pattern and timing of brain metastases among patients with advanced breast cancer treated with trastuzumab. Acta Oncol.

[42] Omuro A.M., Kris M.G., Miller V.A., Franceschi E., Shah N., Milton D.T., Abrey L.E. (2005). High incidence of disease recurrence in the brain and leptomeninges in patients with nonsmall cell lung carcinoma after response to gefitinib. Cancer.

[43] Ruppert A.M., Beau-Faller M., Neuville A., Guerin E., Voegeli A.C., Mennecier B., Legrain M., Molard A., Jeung M.Y., Gaub M.P. (2009). Egfr-tki and lung adenocarcinoma with cns relapse: Interest of molecular follow-up. Eur. Respir. J..

[44] Palmieri D., Chambers A.F., Felding-Habermann B., Huang S., Steeg P.S. (2007). The biology of metastasis to a sanctuary site. Clin. Cancer Res.

[45] Grommes C., Oxnard G.R., Kris M.G., Miller V.A., Pao W., Holodny A.I., Clarke J.L., Lassman A.B. (2011). “Pulsatile” high-dose weekly erlotinib for cns metastases from egfr mutant non-small cell lung cancer. Neuro Oncol.

[46] Kim S., Kim J., Park E., Lin Q., Langley R.R., Maya M., He J., Kim S., Weihua Z., Balasubramanian K. (2011). Astrocytes upregulate survival genes in tumor cells and induce protection from chemotherapy. Neoplasia.

[47] Régina A., Demeule M., Laplante A., Jodoin J., Dagenais C., Berthelet F., Moghrabi A., Béliveau R. (2001). Multidrug resistance in brain tumors: Roles of the blood-brain barrier. Cancer Metast. Rev.

[48] Joo K.M., Song S., Park K., Kim M., Jin J., Bong G., Jang M., Lee G., Kim M., Nam D. (1992). Response of brain specific microenvironment to p-glycoprotein inhibitor: An important factor determining therapeutic effect of p-glycoprotein inhibitor on brain metastatic tumors. Int. J. Oncol.

[49] Heyn C. (2006). *In vivo* mri of cancer cell fate at the single-cell level in a mouse model of breast cancer metastasis to the brain. Magn. Reson. Med.

[50] Serres S., Soto M., Hamilton A., McAteer M., Carbonell W., Robson M., Ansorge O., Khrapitchev A., Bristow C., Balathasan L. (2012). Molecular mri enables early and sensitive detection of brain metastases. Proc. Natl. Acad. Sci. USA.

[51] Berger J.C., Vander Griend D.J., Robinson V.L., Hickson J.A., Rinker-Schaeffer C.W. (2005). Metastasis suppressor genes: From gene identification to protein function and regulation. Cancer Biol. Ther.

[52] Townson J.L., Chambers A.F. (2006). Dormancy of solitary metastatic cells. Cell Cycle.

[53] Nash K.T., Phadke P.A., Navenot J.M., Hurst D.R., Accavitti-Loper M.A., Sztul E., Vaidya K.S., Frost A.R., Kappes J.C., Peiper S.C. (2007). Requirement of kiss1 secretion for multiple organ metastasis suppression and maintenance of tumor dormancy. J. Natl. Cancer Inst.

[54] Hedley B.D., Chambers A.F. (2009). Tumor dormancy and metastasis. Adv. Cancer Res.

[55] McGowan P.M., Kirstein J.M., Chambers A.F. (2009). Micrometastatic disease and metastatic outgrowth: Clinical issues and experimental approaches. Future Oncol.

[56] Goss P.E., Chambers A.F. (2010). Does tumour dormancy offer a therapeutic target?. Nat. Rev. Cancer.

[57] Bragado P., Sosa M.S., Keely P., Condeelis J., Aguirre-Ghiso J.A. (2012). Microenvironments dictating tumor cell dormancy. Recent Res. Cancer.

[58] Sosa M.S., Avivar-Valderas A., Bragado P., Wen H.C., Aguirre-Ghiso J.A. (2011). Erk1/2 and p38alpha/beta signaling in tumor cell quiescence: Opportunities to control dormant residual disease. Clin. Cancer Res.

[59] Kim M.Y., Oskarsson T., Acharyya S., Nguyen D.X., Zhang X.H., Norton L., Massague J. (2009). Tumor self-seeding by circulating cancer cells. Cell.

[60] Troester M.A., Lee M.H., Carter M., Fan C., Cowan D.W., Perez E.R., Pirone J.R., Perou C.M., Jerry D.J., Schneider S.S. (2009). Activation of host wound responses in breast cancer microenvironment. Clin. Cancer Res.

[61] Husemann Y., Geigl J.B., Schubert F., Musiani P., Meyer M., Burghart E., Forni G., Eils R., Fehm T., Riethmuller G. (2008). Systemic spread is an early step in breast cancer. Cancer Cell.

[62] Steeg P.S., Bevilacqua G., Kopper L., Thorgeirsson U.P., Talmadge J.E., Liotta L.A., Sobel M.E. (1988). Evidence for a novel gene associated with low tumor metastatic potential. J. Natl. Cancer Inst.

[63] Horak C.E., Lee J.H., Marshall J.C., Shreeve S.M., Steeg P.S. (2008). The role of metastasis suppressor genes in metastatic dormancy. APMIS.

[64] Shoushtari A.N., Szmulewitz R.Z., Rinker-Schaeffer C.W. (2011). Metastasis-suppressor genes in clinical practice: Lost in translation?. Nat. Rev. Clin. Oncol.

[65] Miller K.D., Weathers T., Haney L.G., Timmerman R., Dickler M., Shen J., Sledge G.W. (2003). Occult central nervous system involvement in patients with metastatic breast cancer: Prevalence, predictive factors and impact on overall survival. Ann. Oncol..

[66] Gabos Z., Sinha R., Hanson J., Chauhan N., Hugh J., Mackey J.R., Abdulkarim B. (2006). Prognostic significance of human epidermal growth factor receptor positivity for the development of brain metastasis after newly diagnosed breast cancer. J. Clin. Oncol.

[67] Lin N.U., Bellon J.R., Winer E.P. (2004). Cns metastases in breast cancer. J. Clin. Oncol.

[68] Palmieri D., Bronder J.L., Herring J.M., Yoneda T., Weil R.J., Stark A.M., Kurek R., Vega-Valle E., Feigenbaum L., Halverson D. (2007). Her-2 overexpression increases the metastatic outgrowth of breast cancer cells in the brain. Cancer Res.

[69] Clevers H., Nusse R. (2012). Wnt/beta-catenin signaling and disease. Cell.

[70] Lin N.U., Dieras V., Paul D., Lossignol D., Christodoulou C., Stemmler H.J., Roche H., Liu M.C., Greil R., Ciruelos E. (2009). Multicenter phase ii study of lapatinib in patients with brain metastases from her2-positive breast cancer. Clin. Cancer Res.

[71] Kodack D.P., Chung E., Yamashita H., Incio J., Duyverman A.M., Song Y., Farrar C.T., Huang Y., Ager E., Kamoun W. (2012). Combined targeting of her2 and vegfr2 for effective treatment of her2-amplified breast cancer brain metastases. Proc. Natl. Acad. Sci. USA.

[72] Kaplan M.A., Isikdogan A., Koca D., Kucukoner M., Gumusay O., Yildiz R., Dayan A., Demir L., Geredeli C., Kocer M. (2013). Clinical outcomes in patients who received lapatinib plus capecitabine combination therapy for her2-positive breast cancer with brain metastasis and a comparison of survival with those who received trastuzumab-based therapy: A study by the anatolian society of medical oncology. Breast Cancer.

[73] Nguyen D.X., Bos P.D., Massague J. (2009). Metastasis: From dissemination to organ-specific colonization. Nat. Rev. Cancer.

[74] Nguyen D.X., Massague J. (2007). Genetic determinants of cancer metastasis. Nat. Rev. Genet.

